# Single cell-transcriptomic analysis informs the lncRNA landscape in metastatic castration resistant prostate cancer

**DOI:** 10.1038/s41525-024-00401-3

**Published:** 2024-02-23

**Authors:** Debanjan Saha, Ha X. Dang, Meng Zhang, David A. Quigley, Felix Y. Feng, Christopher A. Maher

**Affiliations:** 1 Medical Scientist Training Program, Washington University in St. Louis, St. Louis, MO USA; 2 Department of Internal Medicine, Washington University in St. Louis, St. Louis, MO USA; 3 Department of Radiation Oncology, University of California at San Francisco, San Francisco, CA, USA; 4 Helen Diller Family Comprehensive Cancer Center, University of California at San Francisco, San Francisco, CA USA; 5 Department of Urology, University of California at San Francisco, San Francisco, CA, USA; 6 Department of Epidemiology & Biostatistics, University of California at San Francisco, San Francisco, CA, USA; 7 Division of Hematology and Oncology, Department of Medicine, University of California at San Francisco, San Francisco, CA, USA

**Keywords:** Prostate cancer, Gene expression

## Abstract

Metastatic castration-resistant prostate cancer (mCRPC) is a lethal form of prostate cancer. Although long-noncoding RNAs (lncRNAs) have been implicated in mCRPC, past studies have relied on bulk sequencing methods with low depth and lack of single-cell resolution. Hence, we performed a lncRNA-focused analysis of single-cell RNA-sequencing data (*n* = 14) from mCRPC biopsies followed by integration with bulk multi-omic datasets. This yielded 389 cell-enriched lncRNAs in prostate cancer cells and the tumor microenvironment (TME). These lncRNAs demonstrated enrichment with regulatory elements and exhibited alterations during prostate cancer progression. Prostate-lncRNAs were correlated with *AR* mutational status and response to treatment with enzalutamide, while TME-lncRNAs were associated with *RB1* deletions and poor prognosis. Finally, lncRNAs identified between prostate adenocarcinomas and neuroendocrine tumors exhibited distinct expression and methylation profiles. Our findings demonstrate the ability of single-cell analysis to refine our understanding of lncRNAs in mCRPC and serve as a resource for future mechanistic studies.

## Introduction

lncRNAs are RNA transcripts longer than 200 nucleotides without evidence of coding potential^1^. Recent work has implicated lncRNAs in various steps of the metastatic cascade with a wide array of mechanisms, such as through transcriptional and epigenetic regulation, as well as sequestration and modification of RNA transcripts or proteins^1^. Moreover, multiple upstream mechanisms have been demonstrated to alter lncRNAs in cancer via somatic alterations, epigenetic and transcriptional regulation, and alternative splicing^1^.

Prostate cancer is the most common malignancy for men in the US^2^. The Androgen Receptor (AR) plays a major role in regulating prostate cancer progression and treatment response and is consequently the target of many pharmacologic agents^3^. However, several lncRNAs have also recently been implicated in the biology of this disease. These include genes such as *DANCR*, *DRAIC*, *PCAT29*, *PCAT19*, and *PCAT14*, all of which have been shown to be regulated by the AR, and are important regulators of prostate cancer cell proliferation, division, migration, and invasion^4–8^. The lncRNAs *PCA3* and *lncRNA-ATB* have also been implicated in epithelial-to-mesenchymal transition causing prostate cancer cells to lose features of epithelial cells such as cell–cell adhesion and polarity and gain more invasive, migratory, and anti-apoptotic properties^4,9^. In addition, *GAS-5* has been described as a lncRNA regulated by the mTOR pathway and controls both cellular apoptosis and the binding of AR to DNA^4^. Hence, lncRNAs are key players in many of the mechanisms associated with tumorigenesis in prostate cancer.

Within prostate cancer, while ~78% of men will be diagnosed with localized disease, 12% and 5% will be diagnosed with regionally disseminated and distant disease, respectively^10^. Metastatic castration-resistant prostate cancer (mCRPC) is an aggressive form of prostate cancer that develops after metastatic spread as well as resistance to androgen ablation and carries a poor prognosis^2^. Various lncRNAs have also been demonstrated to regulate disease processes in mCRPC. These include *PCGEM1* which has been shown to increase expression of *AR* splice variants, *HOTAIR* which leads to increased *AR* expression and may contribute to enzalutamide resistance, and *MALAT1* and *SCHLAP1*, which have been demonstrated to be upregulated in mCRPC and interact with EZH2 and the SWI/SNF complex, respectively, to promote prostate cancer cell invasion^4,11,12^. Thus, while many lncRNAs have emerged recently within the context mCRPC, a vast majority remain uncharacterized and underappreciated in their roles within this disease.

Previous studies have leveraged integrative genomic analyses to elucidate the genomic and epigenomic drivers of mCRPC, such as the occurrence of mutations in the *AR* region, genes associated with *RB1* biallelic inactivation, and the presence of hypomethylated regions in prostate cancer-specific genes^13–15^. In addition, recent studies using single-cell transcriptomics (scRNA-seq) have analyzed genes associated with the treatment of mCRPC with enzalutamide, a commonly used AR signaling inhibitor for patients with late-stage disease^16^. Studies have also analyzed genes associated with small cell neuroendocrine prostate cancer (SCNC), an aggressive histologic variant that arises due to shifts in lineage plasticity during treatment^16^. Lastly, recent work has demonstrated the existence of an immunosuppressive tumor microenvironment (TME) to preclude anti-tumor immunity within mCRPC^17^. Therefore, strategies to integrate multiple “-omics” datasets specific to mCRPC have the potential to also benefit our understanding of lncRNAs in prostate cancer.

Despite the emerging roles of lncRNAs in mCRPC, the existing studies have several limitations. First, many of these studies have focused on individual lncRNAs with few focusing on a comprehensive characterization of the universe of lncRNAs associated with various aspects of this disease^6,7,12^. In addition, all of the systematic studies have relied on bulk RNA-sequencing data, which represents an admixed signal that fails to capture the gene expression heterogeneity between cancer cells and the TME^18,19^. In doing so, such analyses can lead to erroneous conclusions on the processes that are regulated by lncRNAs. Lastly, previous work on lncRNAs in mCRPC has not integrated single-cell expression data with other data modalities such as genomic, epigenomic, and clinicopathologic features to provide insight on the upstream and downstream mechanisms of lncRNA transcriptomic aberrations. Therefore, the goals of this study were to integrate multiple mCRPC-specific datasets with scRNA-seq data to characterize lncRNAs expressed in mCRPC cell types, their association with genomic and regulatory features, and relationship to tumor progression, genomic status, treatment resistance, survival outcomes, and histologic transformation.

## Results

### lncRNAs are enriched in prostate cancer cells and the TME in mCRPC

We sought to identify lncRNAs enriched within cancer cells and the TME in mCRPC based on gene expression by analyzing single-cell RNA-sequencing data from 2170 cells derived from 15 biopsies at bone, lymph node, and liver metastatic sites from 14 mCRPC patients profiled in ref. 16. We reasoned that based on the high average depth (mean depth of 1.5 million reads per cell) of sequencing coupled with the representation of various metastatic sites, treatment histories, and histologic variants, this dataset would provide substantial cell diversity and sequencing coverage for lncRNA analysis in mCRPC and the TME. Accordingly, this scRNA-seq dataset demonstrated expression of ~70% of annotated lncRNAs with lncRNAs exhibiting lower expression values when compared to protein-coding genes (Supplementary Fig. 1a, b).

**Fig. 1:**
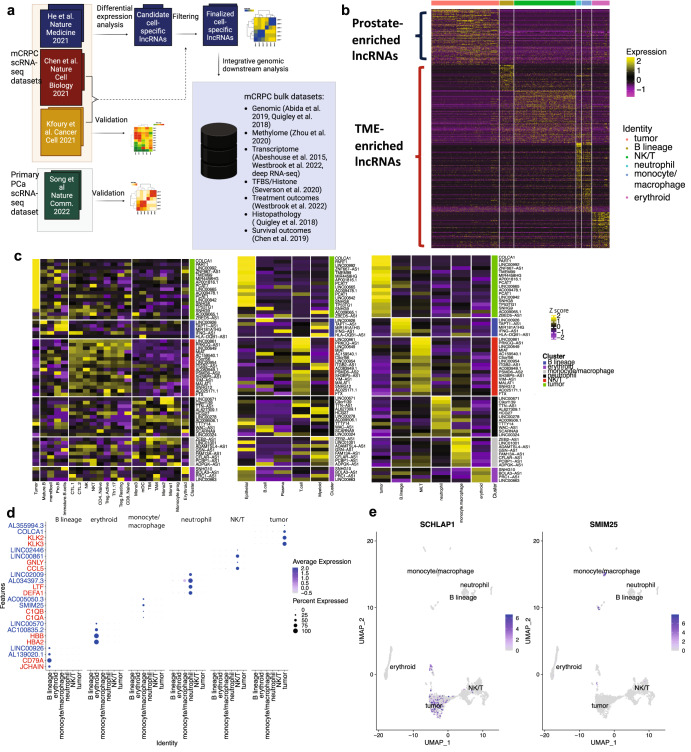
Discovery and validation of prostate and TME-enriched lncRNAs. **a** Schematic of lncRNA discovery and validation from scRNA datasets, followed by integration with mCRPC datasets. **b** Heatmap of Z-scores of log2 TPM expression of prostate (91) and TME (289)-enriched lncRNAs. **c** Validation of common cell-enriched lncRNAs in discovery set from ref. 16 (right) with scRNA-seq datasets from ref. 17 (left) and ref. 20 (middle). lncRNAs and their corresponding clusters are listed as rows on the right side of each plot with cell types labeled as columns on the bottom and are grouped based on cell lineage. Cells in ref. 17 are grouped as such: Tumor as tumor; Mature B, mem B, Pro B, and immature B cells as B lineage; CTL1/2, NK, NKT, CD4 + /CD8+ naive, Treg Active/Inactive, and Th1/17 as NK/T cells; Mono1/2/3, TAM, TIM, mDC, Monocyte progenitor as monocyte/macrophages; and erythroid as erythroid. This dataset did not contain neutrophils. Cells in ref. 20 are grouped as such: Epithelial as tumor; B and Plasma cell as B lineage; T cell as NK/T, and Myeloid as both neutrophil and monocyte/macrophage. This dataset did not contain erythroid cells. **d** Top two enriched lncRNAs in each cell type vs. top two enriched protein-coding genes. **e** UMAP plots of *SCHLAP1* enriched in prostate cancer cells and *SMIM25* enriched in monocyte/macrophages.

To annotate lncRNAs enriched in various cellular compartments within mCRPC, we labeled cells based on the clustering and annotation performed in the original study, which revealed clusters of prostate cancer, monocyte/macrophage, NK/T, B, neutrophil and erythroid cells. Subsequently, a computational pipeline was used to derive lncRNAs associated with each major cell type and filter lncRNAs that demonstrated low expression and poor cell specificity (“Methods”, Fig. 1a). This yielded 389 cell-enriched lncRNAs of which 91 were enriched in prostate cancer cells (pca-lncRNAs) and 298 were enriched in the TME cells (tme-lncRNAs) (Fig. 1b and Supplementary Table 1). These candidates were validated using two additional scRNA-seq datasets, one from 9 bone lesions from metastatic CRPC patients in ref. 17 and another from 11 tumors from primary prostate cancer patients in refs. 17,20. In total, 61 genes from our list of cell-enriched lncRNAs were expressed in these datasets and upon grouping of cell types, we found similar trends in cell specificity thereby validating many of these identified lncRNAs (Fig. 1c). We also compared the cell specificity of the top enriched lncRNAs with protein-coding genes within the original scRNA-seq dataset using similar criteria for cell enrichment, which demonstrated that the most enriched lncRNAs had comparable cell specificity to the most enriched protein-coding genes (Fig. 1d). We also identified lncRNAs that were known to be cell-type specific including *SCHLAP1* enriched in prostate cancer cells (Fig. 1e, avg_log2FC = 4.3, FDR = 5.6 × 10^−73^) and *SMIM25* enriched in monocyte/macrophage cells (Fig. 1e, avg_log2FC = 6.71, FDR = 1.7 × 10^−137^)^18,21^. In summary, our analysis of single-cell transcriptomic data revealed cell-enriched lncRNAs in mCRPC, validated across independent datasets, that could be used for further characterization.

### mCRPC-associated lncRNAs have a distinct genomic and regulatory landscape

**Fig. 2:**
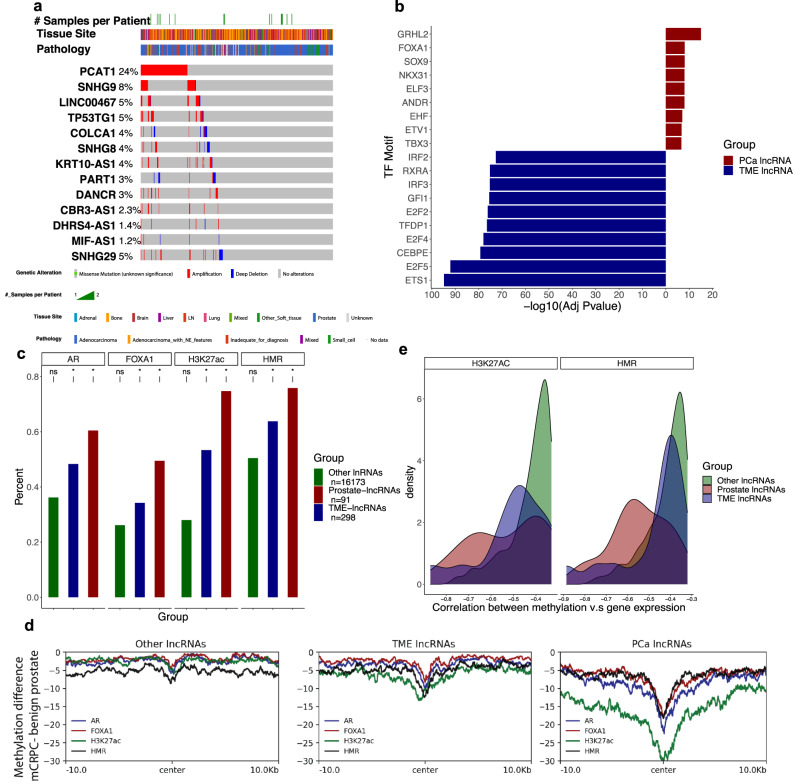
Epigenomic features are enriched and functionally important in mCRPC-associated lncRNAs. **a** Mutational landscape of pca-lncRNAs with at least one somatic mutation in mCRPC in refs. 22–24. **b** Transcription factor binding site (TFBS) motifs in pca- (*n* = 91) and tme- (*n* = 289) lncRNAs after filtering for protein-coding genes in prostate/TME cell types. **c** Enrichment of overlap for AR, FOXA1, and H3K27ac ChIP-seq and HMRs in prostate (*n* = 91), TME (*n* = 289), and remaining lncRNAs in hg38 (*n* = 16173).”*” denotes significant overlap and “ns” denotes nonsignificant overlap^14,26^. **d** Sum of methylation differences of regulatory elements in prostate, TME, and remaining lncRNAs in hg38 downsampled to 91 genes between mCRPC and benign prostate^14^. **e** Correlation of H3K27ac/HMR methylation and RNA expression in prostate (*n* = 28), TME (*n* = 27), and remaining lncRNAs in hg38 (*n* = 439) with statistically significant associations^14^.

After identifying lncRNAs strongly associated with prostate cancer cells in mCRPC, we investigated genomic aberrations in these genes to assess the contribution of somatic mutations in lncRNA alterations. We found 13/91 (14.3%) pca-lncRNAs had at least one somatic mutation using whole-genome sequencing data from a cohort of 444 mCRPC patients in refs. 22–24. Most genes exhibited copy number variants (CNVs) at low frequencies (mean = 5.4%) in this cohort, with the exception of *PCAT1* being amplified in 24% of patients in this study (Fig. 2a). A similar trend was noted for pca-lncRNAs in TCGA primary prostate cancer data from ref. 25 as well (Supplementary Fig. 2a). While genomic amplification could contribute to the over-expression of *PCAT1* in mCRPC, studies have also attributed this to co-amplifications with the nearby oncogene *MYC*^13^. Analysis of tme-lncRNAs revealed similarly low frequencies of CNVs with the exception *PVT1*, which again can be attributed to its proximity with *MYC* amplifications (Supplementary Fig. S2b).

Due to the non-focal nature of CNVs and the paucity of mutations targeting lncRNAs, we reasoned that changes in the regulatory landscape may better explain the differences in expression of cell-enriched lncRNAs. To evaluate this hypothesis, we sought to first characterize the regulatory landscape of cell-enriched lncRNAs. We first extracted promoter sequences for pca- and tme-lncRNAs and performed a differential motif search to identify putative transcription factor binding sites enriched in either prostate or TME cells, followed by filtering for transcription factor genes enriched in these respective cell types. This analysis identified several known transcription factors motifs in pca-lncRNAs and implicated in mCRPC biology, such as AR, FOXA1, NKX3.1 and the ETS family of proteins (Fig. 2b). Next, regulomic data derived from mCRPC biopsies profiled by Zhao et al.^14^ and Severson et al.^26^, such as ChIP-sequencing data for AR and FOXA1 transcription factor binding sites as well as H3K27ac peaks and hypomethylated regions (HMRs), which denote regulatory elements, was assessed for overlap with cell-enriched lncRNAs. We found a strong, significant enrichment for pca-lncRNAs (AR: 60.4%, FOXA1: 49.5%, H3K27ac: 74.7%, HMR: 75.8%,) and a weaker enrichment for tme-lncRNAs (AR: 48.3%, FOXA1: 34.2%, H3K27ac: 53.4%%, HMR: 63.8%) overlapping these regulatory features (Fig. 2c). In addition, we analyzed methylation profiles from mCRPC and benign prostate tissue for these regions near transcriptional start sites. This demonstrated a large decrease in methylation for regulatory elements in pca-lncRNAs (minimum total methylation difference = −30%) when compared to tme-lncRNAs (minimum total methylation difference = −13.3%) and a background set of lncRNAs (minimum total methylation difference = −8.65%, Fig. 2d). A similar trend was also noted for cell-specific lncRNAs between mCRPC and primary prostate cancers (Supplementary Fig. 2c). The correlation between methylation and gene expression at H3K27ac and HMR regions showed the largest anticorrelation for pca-lncRNAs (H3K27ac: mean Pearson correlation = −0.53; HMR: mean Pearson correlation = −0.55) and a weaker anticorrelation for tme-lncRNAs lncRNAs (H3K27ac: mean Pearson correlation = −0.50; HMR: mean Pearson correlation = −0.47) (Fig. 2e). Lastly, we used EpiMap data of H3K27ac profiling from various cell lines to characterize regulatory elements identified in mCRPC^27^. H3K27ac peaks near tme-lncRNAs showed strong enrichment in data derived from epithelial, B- and T-cell lines, which is consistent with the role for these lncRNAs in immune cells (Supplementary Fig. S2d). In contrast, peaks near pca-lncRNAs were highly enriched in epithelial, reproductive and gastrointestinal cell lines, which is a similar finding as demonstrated in prior literature (Supplementary Fig. S2d)^28^. Taken together, these results indicate the presence of key regulatory elements, namely transcription factor binding sites, histone acetylation peaks, and HMRs, that are enriched and functionally important for dictating gene expression primarily of pca-lncRNAs.

### lncRNAs exhibit cell-specific expression changes during tumor progression

**Fig. 3:**
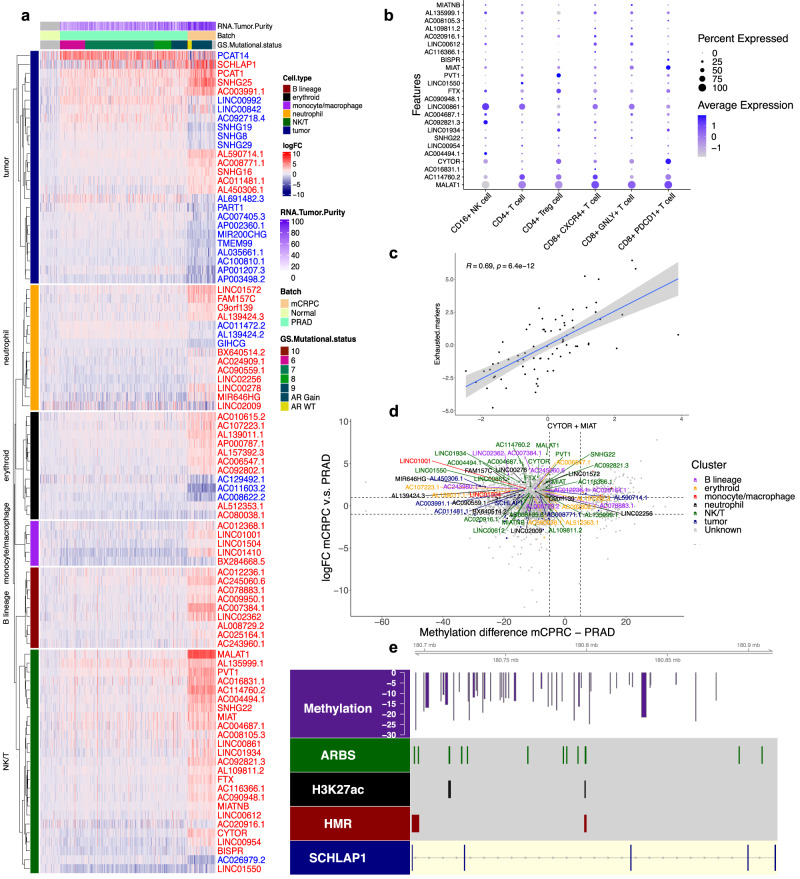
Single-cell analysis informs bulk expression analysis of lncRNAs associated with prostate cancer progression. **a** Heatmap of differentially expressed lncRNAs in bulk RNA-seq for *n* = 333 primary prostate and *n* = 74 mCRPC samples with significantly upregulated genes in red and downregulated genes in blue^25^. Cell types associated with each gene are shown on the left side. Samples are ordered column-wise with normal adjacent prostate, primary prostate cancer, and mCRPC from left to right. Samples in primary prostate cancers are ordered by increasing Gleason Score and for mCRPC by the genomic status of the *AR* region. **b** Dot plot of differentially expressed NK/T-lncRNAs and T-cell subtypes in scRNA-seq data. **c** Correlation between expression of *MIAT* and *CYTOR* with exhausted T-cell markers from in ref. 16 in bulk mCRPC RNA-seq data^16^. **d** Methylation of differentially methylated regions (*x* axis) nearby differentially expressed (*y* axis) lncRNAs in mCRPC vs. primary prostate cancer^14^. **e** Gene plot of *SCHLAP1* overlaid with HMRs (red), H3K27ac (black), AR binding sites (green), and differentially methylated regions (DMRs) (purple) between mCRPC vs. primary prostate cancer^14,26^.

To assess lncRNAs associated with prostate cancer progression, we performed differential expression analysis comparing bulk RNA-Seq data between primary prostate cancers in ref. 25 (TCGA, *n* = 333)^25^ and mCRPC (*n* = 74). Differentially expressed lncRNAs were subsequently categorized as either enriched in prostate cancer cells or TME cells based on the aforementioned single-cell analysis. This revealed several known and underappreciated lncRNAs that have significantly altered expression during tumor progression in a cell-specific fashion (Fig. 3a and Supplementary Table 2). For example, we found the pca-lncRNAs *SCHLAP1* and *PCAT14*, both known to be enriched in prostate cancer cells, to exhibit upregulation (logFC = 1.28, FDR = 1.63 × 10^−2^) and downregulation (logFC = −3.77, FDR = 1.83 × 10^−20^), respectively, in metastases compared to primary tumors. This recapitulates similar findings for these lncRNAs in previous studies^6,12^. Several underrecognized associations were also detected such as an increase in NK/T-cell-enriched lncRNAs *MIAT* (logFC =2.1, FDR = 1.94 × 10^−25^) and *CYTOR* (logFC = 3.33, FDR = 2.86 × 10^−84^) in mCRPC samples. Based on further sub-setting of single cells from mCRPC biopsies, these genes were found to be enriched in CD8 + PDCD1 + T cells among NK/T-cell lncRNAs (Fig. 3b). Further supporting this, a strong positive correlation was observed in bulk sequencing data between *MIAT* and *CYTOR* expression with genes associated with a dysfunctional T-effector cell phenotype (Pearson correlation 0.66, *P* = 2.2 × 10^−10^, Fig. 3c). This result is consistent with an earlier study implicating these lncRNAs in immune infiltration within the TME^29^. Likewise, our study associates these genes with immune checkpoint signaling in prostate cancer due to the integration of single-cell analysis^29^.

To further explore potential mechanisms of lncRNA dysregulation during prostate cancer progression, we annotated differentially expressed lncRNAs with nearby differentially methylated regions identified in a prior report from ref. 14 between mCRPC and primary prostate cancer^14^. A majority of genes that were upregulated in metastases were also found to have decreased levels of CpG methylation nearby (Fig. 3d), suggesting methylation could be major mechanism of lncRNA regulation in mCRPC. Several of these included both pca-lncRNAs as well as tme-lncRNAs, suggesting that a variety of cell-enriched lncRNAs exhibit alterations in methylation as a result of tumor progression. For example, *SCHLAP1* was found to contain decreased regions of methylation near regulatory elements for transcription factor binding, which may explain its upregulation in the metastatic setting (Fig. 3e). Taken together, via integration of multi-omic bulk and single-cell transcriptomic data we delineated cell-specific changes in lncRNA expression and a potential link to methylation during prostate cancer progression.

### Prostate-enriched lncRNAs are associated with *AR* amplifications and are correlated with enzalutamide treatment

**Fig. 4:**
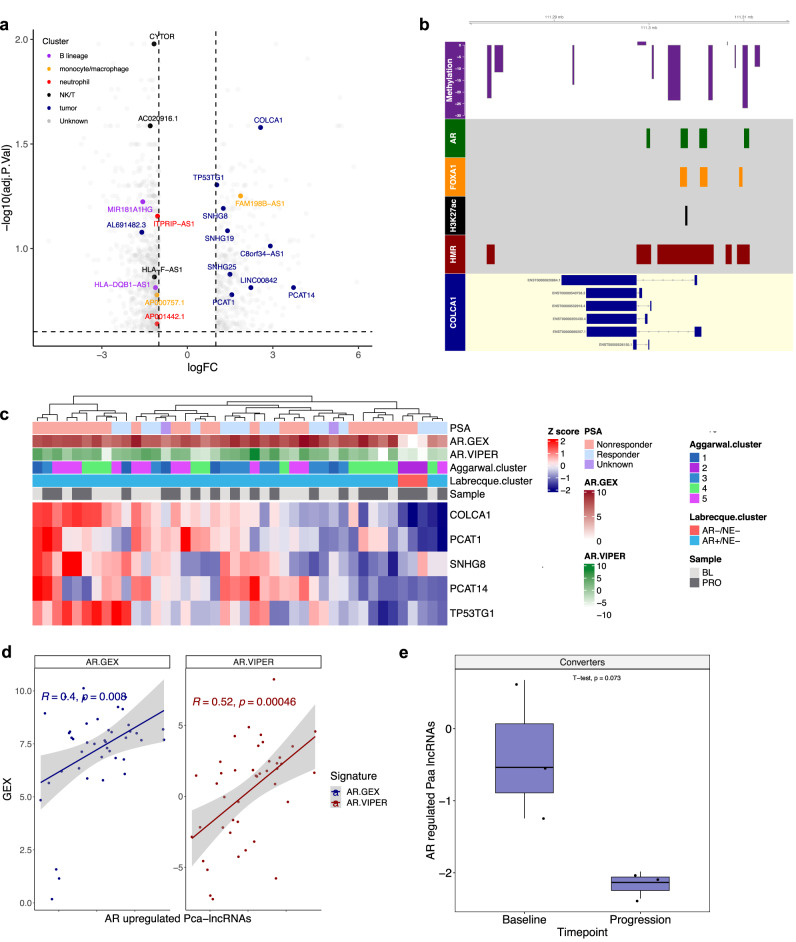
Pca-lncRNAs are associated with *AR* amplifications and treatment with enzalutamide. **a** Volcano plot of differentially expressed lncRNAs in tumors with *AR* region amplifications v/s wild-type in bulk RNA-seq data from *n* = 64 mCRPCs^16^. **b** Gene plot of *COLCA1* with HMRs (red), H3K27ac (black), AR (green) and FOXA1 (yellow) binding sites, and DMRs (purple) in mCRPC vs. benign prostate^14,26^. **c** Heatmap of AR-upregulated pca-lncRNAs in baseline and progression biopsies for matched patient samples in laser-capture microdissected (LCM) RNA-seq data^13,30^. **d** Correlation of AR-upregulated pca-lncRNAs with *AR* gene expression (blue) and AR activity (red) in LCM RNA-seq data^30^. **e** Expression of AR-upregulated pca-lncRNAs in LCM RNA-seq data for AR + /NE- to AR-/NE- converters (*n* = 3) with paired *T* test *P* value above^30^.

The AR is a major driver of prostate cancer progression and treatment response and AR signaling inhibitors are commonly used for patients with mCRPC^30,31^. Thus, we sought to understand the role of AR signaling in lncRNA expression. We performed differential expression analysis of bulk RNA-seq data between mCRPC biopsies with *AR* region amplifications relative to wild-type tumors determined via whole-genome sequencing from ref. 13. This revealed several lncRNAs upregulated with *AR* amplifications with many of them enriched in prostate cancer cells (Fig. 4a and Supplementary Table 3). When applying this methodology to protein-coding genes in this dataset, we recovered many established AR-upregulated pca- protein-coding genes such as *SLC45A3* and *TMEFF2* (Supplementary Fig. 3a)^14^. Our results also recapitulated known AR-upregulated pca-lncRNAs such as *PCAT14* (Fig. 4a)^32^. Moreover, this analysis yielded AR-upregulated pca-lncRNAs, such as *COLCA1*, which has only recently been implicated in prostate cancer^33^. Analysis of *COLCA1* at the gene level demonstrated several AR and FOXA1 binding sites within regulatory elements near its gene body, suggesting its regulation by AR and AR co-regulators (Fig. 4b). Similarly, another example of an underappreciated lncRNAa that potentially contributes to mCRPC is *TP53TG1*. This pca-lncRNA was upregulated in mCRPCs with AR amplifications and was downregulated upon enzalutamide treatment. Furthermore, its promoter contains multiple regulatory elements and AR binding sites (Supplementary Fig. S3b). Prior literature has demonstrated an oncogenic role for *TP53TG1* in pancreatic ductal adenocarcinoma, retinoblastoma, and nasopharyngeal carcinoma^34–36^, and future work will be needed to validate the function of this lncRNA in mCRPC.

Using our list of AR-upregulated pca-lncRNAs, we next sought to validate the association of androgen signaling with pca-lncRNA expression and further explore this relationship in the context of AR directed therapies. To do this, we analyzed data from a recently published RNA-seq dataset with biopsies taken from 21 mCRPC patients at baseline and during progression with enzalutamide profiled in refs. 30,31. Although the number of lncRNAs profiled in these studies was limited, we found that expression of AR-upregulated pca-lncRNAs that were measured in this dataset, including *PCAT14* and *COLCA1*, varied across patient biopsies with three major clusters containing either high, moderate, or low expression of these genes, respectively (Fig. 4c). These genes were subsequently found to contain positive correlations with both *AR* gene expression (Pearson correlation=0.4) and AR regulon activity (Pearson correlation = 0.52) as measured by the VIPER algorithm in the original studies (Fig. 4d)^30^. Based on this association with AR signaling, we reasoned that patients who responded to enzalutamide treatment (defined by a 50% decrease in PSA at 12 months in the original studies) should downregulate the expression of AR-upregulated pca-lncRNAs when compared to patients who failed to respond to treatment. We observed a trending decrease in pca-lncRNA expression (paired *T* test *P* value = 0.057, Supplementary Fig. 3c) and a significant decrease in AR-upregulated protein-coding gene expression (paired *T* test *P* value = 0.0017, Supplementary Fig. 3d) in patients responding to treatment.

These studies also identified three patients whose tumors developed treatment resistance due to alterations in lineage plasticity from an AR + /NE− phenotype to an AR-/NE- status, suggesting alterations in AR target gene expression. Using our methodology, expression of AR-upregulated pca-lncRNAs (paired *T* test *P* value = 0.073, Fig. 4e) showed a strong trend towards decreased expression, which was similar to the findings with AR-upregulated pca-protein-coding genes (paired *T* test *P* value = 0.22, Supplementary Fig. 3e) and the AR VIPER score (paired *T* test *P* value = 0.13, Supplementary Fig. 3f) from the original studies^30^, suggesting that lineage plasticity due to treatment resistance may also alter the landscape of AR-regulated lncRNAs. In summary, we found that *AR* amplifications are strongly associated with expression of pca-lncRNAs, which led to the identification of androgen-regulated lncRNAs. Moreover, these genes were found to be associated with response to enzalutamide and may exhibit alterations during lineage shifts as a result of treatment resistance.

### TME-lncRNAs are enriched in *RB1* loss tumors and are associated with poor overall survival

**Fig. 5:**
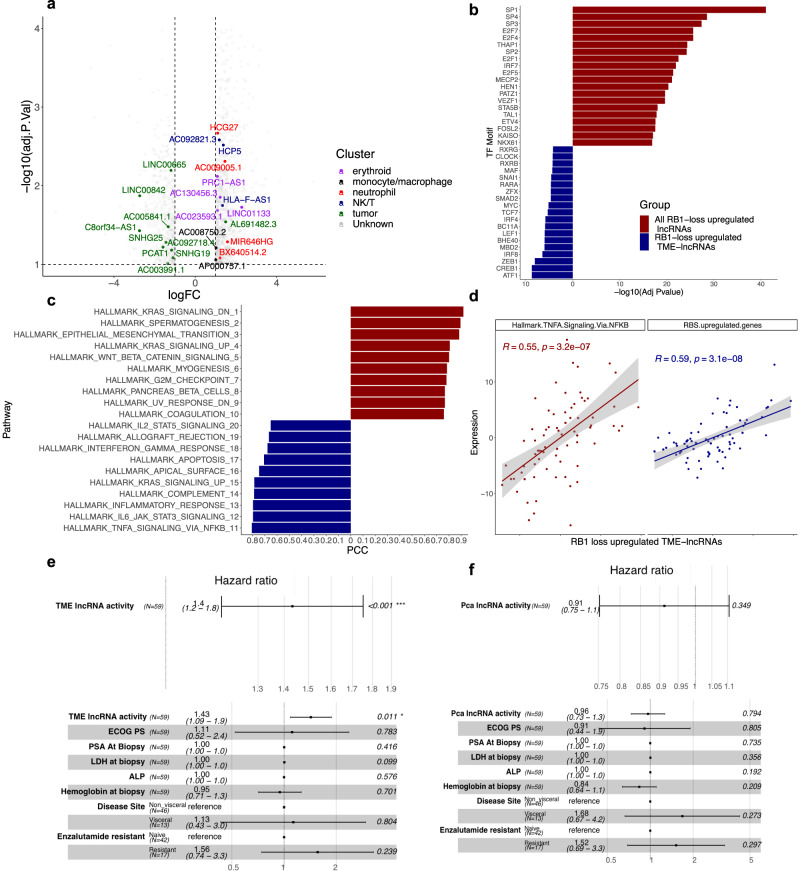
TME-lncRNAs are upregulated in tumors with RB1 loss and are associated with poor outcomes. **a** Differential expression analysis of lncRNAs in *n* = 64 bulk RNA-seq mCRPC biopsies between samples with biallelic inactivation of *RB1* vs. monoallelic/wild-type *RB1*^13^. Genes are highlighted based on their enrichment in cell types from single-cell analysis. **b** Motif enrichment for all upregulated lncRNAs (red) and tme-lncRNAs (blue) in *RB1*-deleted tumors. **c** Hallmark MSigdb pathways correlated with all upregulated lncRNAs (red) and tme-lncRNAs (blue) in *RB1*-deleted tumors from scRNA-seq data. **d** Correlation of upregulated tme-lncRNAs with the Hallmark TNFA Signaling pathway and the upregulated genes in the RBS signature from ref. 15. **e** Forest plots of univariate (top) and multivariate (bottom) analysis of *n* = 59 mCRPC samples with bulk RNA-seq for *RB1* loss upregulated tme-lncRNAs and overall survival. **f** Forest plots of univariate (top) and multivariate (bottom) analysis of *n* = 59 mCRPC samples with bulk RNA-seq of *RB1* loss downregulated pca-lncRNAs and overall survival.

Previous studies have found mutations in the tumor suppressor gene *RB1* to be associated with poor overall survival across multiple tumor types and within mCRPC^15,37–40^. Moreover, prognostic signatures have been defined to detect genes that exhibit transcriptomic perturbations in tumors with *RB1* deficiency, but these have not focused on the role of lncRNAs^15^. To define lncRNAs associated with *RB1* loss, we performed differential expression analysis of bulk RNA-seq data between mCRPC biopsies with biallelic inactivation of *RB1* relative to monoallelic and wild-type tumors determined via whole-genome sequencing from ref. 13 (Supplementary Table 4). Upon annotating lncRNAs to their respective cell types, it was found that many upregulated lncRNAs were not enriched in a particular cell type, likely reflecting non-cell-specific proliferative pathways that typically increase upon *RB1* inactivation. However, a subset of upregulated lncRNAs were annotated as mostly immune cell-specific tme-lncRNAs, suggesting a link to immune infiltration in *RB1* deficient tumors (Fig. 5a). In contrast, downregulated genes consisted of pca-lncRNAs associated with androgen signaling. Similar findings were found for protein-coding genes in this dataset (Supplementary Fig. 4a).

For downstream analysis, we focused on upregulated tme-lncRNAs enriched in components of the immune system (NK/T, monocyte/macrophage, neutrophil, B lineage cells) and removed erythroid progenitor-enriched lncRNAs due to their tendency to overlap with genes associated with proliferative pathways. Differential motif analysis found that lncRNAs upregulated in *RB1* loss tumors showed enrichment for E2F/SP1 transcription factors (SP1 FDR = 3.2 × 10^−18^, E2F4 FDR = 9.8 × 10^−10^), consistent with the role of RB1 in cell cycle regulation. However, immune-specific tme-lncRNAs were enriched for motifs specific to immune cells, such as the IRF family of transcription factors (IRF8 FDR = 1.6 × 10^−3^, Fig. 5b). Moreover, pathway analysis in scRNA-seq data revealed that this subset of genes was highly correlated with immune and inflammatory pathways (Hallmark TNFA signaling Pearson correlation=0.80) and distinct from the pathways seen with the entire gene set, which consisted of epithelial-to-mesenchymal transition (Pearson correlation = 0.88), G2M (Pearson correlation=0.78) and E2F (Pearson correlation = 0.72) signatures classically associated with *RB1* deficiency (Fig. 5c)^15^. Similar results were also seen for protein-coding genes and their corresponding pathways associated with *RB1* deletions (Supplementary Fig. 4b). This association with immune and inflammatory signatures was also seen using bulk RNA-seq data (Hallmark TNFA signaling Pearson correlation = 0.55, Fig. 5d). Furthermore, we compared the expression of this subset of tme-lncRNAs with a previously published tumor-intrinsic signature of genes associated with *RB1* loss and poor outcomes from ref. 15. We found a strong positive correlation between these two gene sets, suggesting that *RB1* loss-associated genes are correlated with reciprocal changes in the immune microenvironment of mCRPCs (Pearson correlation = 0.59, Fig. 5d).

To understand the contribution of cell-specific lncRNAs towards the prognostic relevance of this existing signature of *RB1* loss, we tested the association of signature activity of these tme-lncRNAs with overall survival in mCRPC patients using outcomes data from ref. 15 (“Methods”). This demonstrated a statistically significant relationship in both univariate and multivariate analysis of poor overall survival with increased tme-lncRNA signature activity (univariate: HR = 1.4, CI = 1.1–1.8, *P* value = 0.001; multivariate: HR = 1.43; CI = 1.09–1.9; *P* value = 0.011 Fig. 5e). Notably, we confirmed that protein coding genes upregulated in *RB1* loss and enriched in the TME also associated with poor patient outcome (Supplementary Fig. 4c). Further, this association with poor outcomes was not observed for pca-lncRNAs or pca-protein-coding genes that were downregulated in *RB1* loss tumors in both uni- and multi-variable analysis (Univariate: HR = 0.91, CI = 0.75–1.1, *P* value = 0.349; Multivariate: HR = 0.96. CI = 0.73–1.3. *P* value = 0.794 Fig. 5f; Supplementary Fig. 4d). Therefore, our findings suggest that specifically immunologic pathways, as detected by cell-enriched lncRNAs, are correlated with established transcriptomic aberrations in *RB1*-deleted tumors and contributes to the association of these genes with poor overall survival, highlighting the importance of the TME in mCRPCs with this genomic alteration.

### Histologic transformation induces expression of lncRNAs in prostate cancer cells

**Fig. 6:**
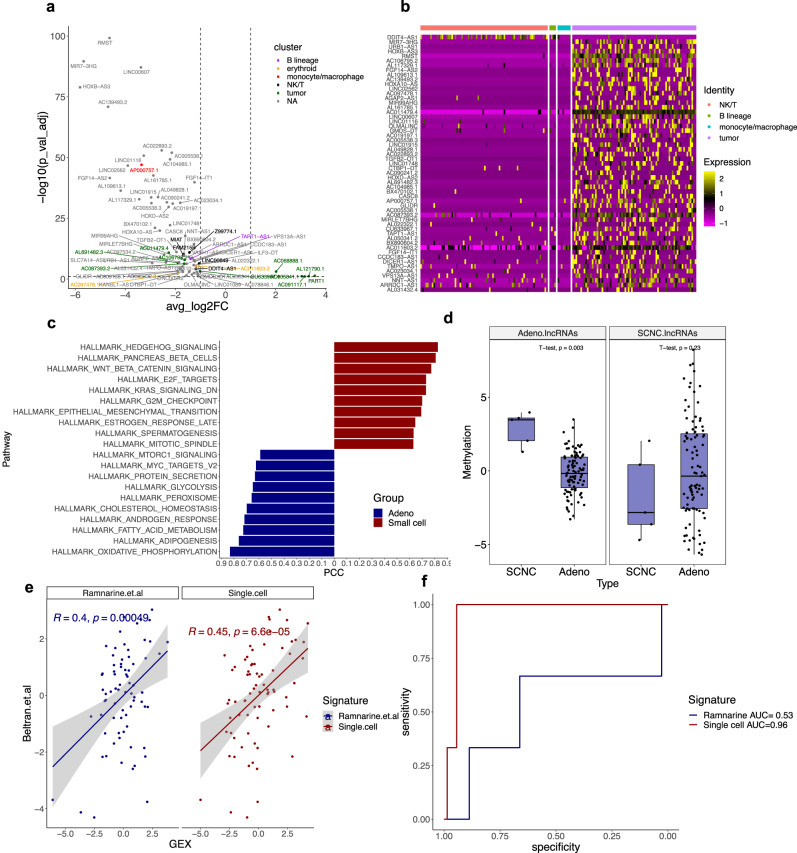
Small cell neuroendocrine prostate cancers express unique prostate-enriched lncRNAs. **a** Differential expression analysis of lncRNAs in adenocarcinomas vs. small cell neuroendocrine (SCNC) carcinomas in scRNA-seq^16^. **b** Heatmap for expression of SCNC-lncRNAs in SCNC cells. **c** Hallmark MSigDB Pathways associated with adeno- and SCNC-lncRNAs in scRNA-seq. **d** Methylation for HMRs near SCNC-lncRNAs and adeno-lncRNAs in *n* = 5 SCNC vs. *n* = 95 adenocarcinoma samples with paired *T* test *P* value above^14^. **e** Correlation of ref. 19 and single-cell-derived SCNC/adeno-lncRNA signatures with neuroendocrine markers from ref. 43. **f** ROC curve analysis of ref. 19 and single-cell-derived SCNC/adeno-lncRNA signatures for tumor histology in *n* = 71 adenocarcinoma and *n* = 3 NEPC bulk RNA-seq samples. AUC values are given on the right-hand side.

Given the presence of a small cell neuroendocrine carcinoma (SCNC) biopsy in the He et al. dataset, we investigated the differences in lncRNA expression between small cell carcinomas and prostatic adenocarcinomas^16^. Differential expression analysis using this dataset revealed multiple genes upregulated in the SCNC sample that could not be assigned to a cell type based on our current list of cell-specific lncRNAs (Fig. 6a and Supplementary Table 5). Analysis of these genes solely in the SCNC sample revealed that most are unique to prostate cancer cells and were likely overlooked due to the paucity of samples containing SCNC features in this dataset (Fig. 6b). Several genes in this list have established associations with neuroendocrine features, such as the lncRNA *RMST* (avg_log2FC = 4.63, FDR = 1.52 × 10^−100^) in this gene set has been implicated in medullary thyroid cancer, a rare neuroendocrine tumor^41^. *RMST* is upregulated in SCNC cells and its promoter contains several regulatory elements that potentially control its gene expression in mCRPC (Supplementary Fig. 5a).

Subsequent pathway association analysis revealed that SCNC-enriched lncRNAs (SCNC-lncRNAs) were strongly associated with sonic hedgehog signaling (Pearson correlation=0.82), which has been described in the literature^42^, while adenocarcinoma-enriched lncRNAs (adeno-lncRNAs) were associated with androgen signaling (Pearson correlation=0.72, Fig. 5c)^42^. As methylation differences have been known to contribute significantly to neuroendocrine prostate cancer^43^, we examined hypomethylated regions near genes deregulated between the two histologic variants using data from ref. 14 It was subsequently found that while SCNC-lncRNAs displayed similar methylation values across histologic variants (*t* test *P* value = 0.23, Fig. 5d), adeno-lncRNAs showed highly divergent methylation profiles (*t* test *P* value = 0.003, Fig. 5d).

Next, as other groups have developed signatures of neuroendocrine prostate cancer^19,43^, we sought to compare our single-cell-derived signature against these. Using bulk RNA-seq data, we found a significant level of positive correlation between our SCNC-lncRNA signature with an established set of SCNC markers based on protein-coding genes from ref. 43 (Pearson correlation=0.45, Fig. 5e)^43^. This was comparable to a previously published SCNC-lncRNA signature derived without single-cell analysis by Ramnarine et al.^19^ (Pearson correlation=0.40). However, when performing AUC analysis to discriminate histologic variants in a validation cohort of 3 SCNC and 71 CRPC-adenocarcinomas using bulk RNA-seq data, our signature had high sensitivity and specificity for detecting neuroendocrine histology and outperformed the previously published lncRNA signature (Single-cell signature AUC = 0.96, Ramnarine et al. signature AUC = 0.53, Fig. 5f). These findings suggest that our signature of SCNC/adeno-lncRNAs is strongly associated with the underlying biology of SCNC and detects prostate cancer cell-specific shifts in the lncRNA landscape during histologic transformation.

## Discussion

In this study, we used single-cell transcriptomic data from mCRPC biopsies coupled with a computational pipeline to identify cell-enriched lncRNAs within prostate cancer cells and the TME. In doing so, we recovered known and discovered many lncRNAs expressed specifically in prostate cancer or TME cells that had previously been masked by bulk sequencing data in prostate cancer. This allowed us to uncover previously underappreciated associations between these genes and regulatory elements, tumor progression, genomic status, and histopathologic variants.

We believe that this is a unique study in that we perform an mCRPC-specific characterization of the lncRNA landscape, as well as utilize single-cell transcriptomic data to yield insights into the cell-specific nature of lncRNAs in this disease. In this analysis, we identified pca-lncRNAs such as *PART1*, which has been implicated in oncogenesis due to its effects on cell proliferation and invasion^44^. This analysis also identified tme-lncRNAs in mCRPC, such as *SMIM25* in monocytes, which has been shown to be a monocyte-specific marker with studies implicating it in autoimmune diseases and antigen processing and presentation across various tumor types^45,46^. The lncRNA *PVT1* was found to be specific to T cells and studies have shown it to influence metabolic pathways in T cells in the context of autoimmunity, as well as antigen processing and presentation throughout several cancers^46,47^. B-cell-specific lncRNAs included *LINC00926* with literature pointing to its role in B cells through its functions on cell junction, protein kinase, and RAS pathways within the TME^48^. Lastly, we identified *FAM157C* in neutrophils, which has been demonstrated to promote granulocyte proliferation^49^.

In this study, we were also able to map the regulatory landscape in mCRPC-associated lncRNAs, which was crucial since we found evidence of aberrations in regulatory elements due to DNA methylation across prostate cancer progression and histologic variants. Upon coupling the results of our single-cell analysis with bulk RNA-seq data for prostate cancer progression, we were able to map out cell-specific alterations in lncRNA expression that had eluded prior investigations. We found well-described changes in pca-lncRNAs such as downregulation of *PCAT14* and upregulation of *SCHLAP1* in metastatic samples^6,12^. Moreover, we discovered changes in NK/T-lncRNAs in the metastatic setting, with data suggesting an increase in CD8 + PDCD1 + T cells that harbor an exhausted T-cell phenotype based on the expression of *CYTOR* and *MIAT*. Prior work has suggested a role for these lncRNAs in the TME with *MIAT* being demonstrated to correlate with infiltrating immune cells in breast cancer^29^. However, future studies will need to be conducted to better establish the mechanistic role of these lncRNAs in dysfunctional T cells.

Pca-lncRNAs were found to be strongly associated with *AR* region amplifications and AR signaling, which recapitulated previously characterized genes such as *PCAT14*^32^. Our analysis also identified androgen-regulated lncRNAs such as *COLCA1*, which was correlated with *AR* genomic status and contained AR binding sites at nearby regulatory elements, suggesting its potential importance in this disease. Prior work on *COLCA1* has implicated it in susceptibility to colorectal cancer^50^. Moreover, a recent study has also demonstrated AR binding sites in the promoter region of *COLCA1*, but future work will need to establish a role for this gene in prostate cancer^33^. When comparing patient biopsies treated with enzalutamide in laser-capture microdissected RNA-seq data, we found a trending decrease in expression of pca-lncRNAs in responders to treatment as well as in three patients whose tumors converted from an AR + /NE- to an AR-/NE- status. Prior work on these three patients has suggested a role for lineage plasticity with a significant downregulation of AR activity and *AR* gene expression^30^. While it is plausible that AR-regulated lncRNAs should also exhibit a concomitant decrease in expression during lineage plasticity, more samples with deeper sequencing to effectively capture the space of pca-lncRNAs and detect differences at lower expression magnitudes are needed to further establish these findings.

Next, we analyzed lncRNAs associated with *RB1* biallelic inactivation and found many genes to be correlated with proliferative signatures that have been demonstrated in other studies^15^. However, by coupling these genes with our results from single-cell analysis, it was discovered that a subset of genes was enriched in immune cells, suggesting a relatively underexplored role for immune signaling in these tumors. Other studies in ovarian, bladder, and localized prostate cancer have pointed to similar conclusions in that tumors with *RB1* deletions were found to harbor a distinct immune profile due to the presence of tumor-infiltrating lymphocytes^51–54^. This subset of tme-lncRNAs was correlated with a previously published tumor-intrinsic transcriptomic signature of *RB1* loss, suggesting that proliferative pathways in cancer cells may lead to concomitant changes in the immune microenvironment^15^. Furthermore, these lncRNAs were found to be associated with poor overall survival, indicating that immune signaling may partially explain the prognostic value of *RB1* deletions in mCRPC. Hence, our single-cell analysis of lncRNAs adds a further level of understanding to the biological processes, namely immune and inflammatory signaling, that are associated with existing *RB1* loss signatures. Future single-cell analysis of mCRPCs with genomic aberrations in *RB1* can allow for a higher-resolution understanding of the immune microenvironment changes in these tumors.

Our final vignette examined the lncRNA landscape in SCNC and found numerous prostate cancer cell-specific changes in lncRNA expression. The utility of single-cell analysis was evident from the finding that our SCNC/adeno-lncRNA signature had high sensitivity/specificity in discriminating tumor pathologies and was strongly associated with markers of neuroendocrine differentiation and hedgehog signaling. Several genes in our signature have been studied in the context of neuroendocrine differentiation. These include *RMST*, which has been shown to be associated with medullary thyroid cancer, a rare neuroendocrine tumor, and *HOXB-AS3*, which has been demonstrated to exhibit high expression in neuroendocrine cells during spinal cord development^41,55^. Future work will be required to better establish these links between our single-cell-derived lncRNAs and SCNC pathology.

In conclusion, we performed an integrative analysis of single-cell transcriptomic and bulk multi-omic data from mCRPC biopsies to characterize the lncRNA landscape of this disease. Our findings revealed a distinction between pca- and tme-lncRNAs, as well as enrichment of regulatory elements in these genes. Differential gene expression analysis of mCRPCs and primary prostate cancers revealed several known and underrecognized lncRNAs that exhibited cell-specific expression changes with tumor progression as well as alterations in DNA methylation. *AR* region amplifications were found to be associated with pca-lncRNAs, which showed a trending decrease in expression in responders to enzalutamide and may be associated with lineage plasticity during treatment resistance. Tumors with *RB1* biallelic inactivation were found to upregulate expression of TME-enriched lncRNAs, which were correlated with a tumor-intrinsic signature of *RB1* loss and also associated with poor outcomes. Lastly, we discovered SCNC-lncRNAs that were correlated with established neuroendocrine markers and strongly capable of distinguishing prostate cancer histology. Our findings demonstrate the utility of single-cell analysis to refine our understanding of lncRNAs in mCRPC and nominate potential mechanisms of actions based on cell-type enrichment. These results will serve as a resource to guide future mechanistic work to explore the biology of these lncRNAs within their appropriate cell types and contribute to the understanding of lncRNAs in the disease processes underlying mCRPC.

## Methods

### Single-cell RNA-seq prostate cancer datasets

To characterize lncRNAs in mCRPC cancer cells and the tumor microenvironment, we utilized published single-cell transcriptome sequencing data from mCRPC and primary tumors. These included sequencing data from He et al. (lymph node, bone and liver metastases, *n* = 14)^16^, Kfoury et al. dataset (bone metastases, *n* = 9)^17^, Chen et al. dataset (lymph node metastases, *n* = 2)^56^, and Song et al. (primary tumors, *n* = 11)^20^ (Fig. 1a).

### Bulk genomic, transcriptomic, methylome, transcription factor, and clinical outcomes mCRPC datasets

Whole-genome sequencing data were analyzed from ref. 22 (*n* = 444) and ref. 13 (*n* = 101). DNA methylation data (*n* = 100) were obtained from ref. 14 at dbGAP (phs001648) and bulk RNA-sequencing data (*n* = 74) at EGAS00001006275. Overall survival data with clinical covariates for these mCRPC samples was downloaded from ref. 15 (*n* = 100). Laser-capture microdissection RNA-sequencing data with associated metadata was obtained from ref. 30 (*n* = 22). Chromatin Immunoprecipitation (ChIP)-sequencing datasets for AR, FOXA1, and H3K27ac were obtained from ref. 26 (*n* = 4). RNA-sequencing count data from TCGA primary prostate cancer samples (*n* = 333) and normal adjacent tissue (*n* = 50) and the associated metadata were downloaded from ref. 25.

### Discovery and validation of cell-specific lncRNAs in mCRPC and TME

To identify lncRNAs with cell-type-specific expression in mCRPC prostate cancer cells and the TME, we performed differential expression analysis using the Seurat package to compare lncRNA expression across cell types using data from refs. 16,57. TPM normalized data from He et al. were log-transformed and merged with metadata labels. Subsequently, differential gene expression analysis for lncRNAs contained in CellRanger GRCh38-2020-A was performed by using the Seurat package R function FinalAllMarkers and filtered for genes detectable in at least 10% of cells with an adjusted *P* value of less than 10% and average log2 fold change greater than 1. This yielded a candidate list of cell-enriched lncRNAs^57^.

To ensure that cell-enriched lncRNAs identified in ref. 16 were not also enriched in a different cell type not profiled in ref. 16, additional filtering was performed. Two other mCRPC scRNA-seq datasets were used for filtering^16^. Raw count data from ref. 17 were log CPM normalized followed by merging with metadata labels^17^. Similar differential gene expression analysis for lncRNAs was performed on this dataset. For each cell type in the candidate list, cell-enriched lncRNAs were filtered if they were found to be enriched in different cell types in ref. 17 than their original designation. A similar approach was used after downloading marker genes from ref. 56 by filtering and removing cell-enriched lncRNAs corresponding to cell types unique to this dataset^56^. This yielded a final list of cell-enriched lncRNAs including lncRNAs enriched in prostate cancer cells (pca-lncRNAs) and lncRNAs enriched in the TME cells comprising of various immune cells (tme-lncRNAs). A similar approach was also used for protein-coding genes in ref. 16 to derive a final list of cell-enriched protein-coding genes in prostate cancer cells and TME cells.

For orthogonal evidence of cell specificity, we used scRNA-seq data from refs. 17,20. Similar differential expression was performed to identify cell-enriched lncRNAs in the Kfoury et al. and Song et al. datasets. Cell-enriched lncRNAs in these two datasets were used to confirm cell-type-specific expression of the nominated cell-enriched lncRNAs identified in ref. 16. Due to platform differences, only a subset (15.7%) of cell-enriched lncRNAs identified in ref. 16 was profiled in the Kfoury et al. and Song et al. datasets and used for this orthogonal validation^16,26,27^.

### Motif analysis

Promoters for motif analysis of prostate- and TME-enriched lncRNAs were extracted using the promoters function in the EnsDb.Hsapiens.v86 R package^58^. Next, the getSeq function was used in the BSgenome.Hsapiens.UCSC.hg38 R package^59^. Fasta files were uploaded to https://meme-suite.org/meme/tools/centrimo to perform differential motif analysis^60^. Motif enrichment was set to “Anywhere” and the HOCOMOCO Human motif database was used. Motifs in Fig. 2b were further filtered for transcription factor protein-coding genes enriched in prostate and TME cell types using the FindMarkers R function with cutoffs of genes being detectable in at least 10% of cells with an adjusted *P* value of less than 10% and average log2 fold change greater than 1.

### Enrichment of regulatory elements with lncRNAs

For AR, FOXA1, and H3K27ac, ChIP-seq data from ref. 26 was used with replicates being concatenated and merged using bedtools merge^26,61^. UCSC liftover was used to convert coordinates to Hg38^62^. Hypomethylated regions (HMRs) from ref. 14 were also obtained from the supplementary data^14^. Overlap of ChIP-seq and HMR data with lncRNA gene body Hg38 coordinates was performed using the subsetByOverlaps R function and enrichment was assessed using Fisher test followed by FDR correction^63^.

### Methylation profiles of regulatory elements with lncRNAs

The DMR bedgraph file between mCRPC and benign prostate was used from ref. 14 and converted to BigWig format using the UCSC bedGraphtoBigWig tool^62^. Next, ChIP-seq and HMR bed files were subset to include regions within a 20-kilobase window around lncRNA TSSs using the promoters function in the EnsDb.Hsapiens.v86 R package^58^. Deeptools functions ComputeMatrix and PlotProfile were used to plot the sum of DMR methylation differences within this subset of regions for prostate, TME, and a background set of lncRNAs^64^. All sets of lncRNAs were downsampled to 91 to match the number of prostate-enriched lncRNAs and remove any bias due to the size of the gene set.

### Correlation of methylation and expression for lncRNAs

Results from correlation analysis in ref. 14 was obtained from the supplementary data^14^. For both HMRs and H3K27ac regions with significant Spearman correlation between methylation and gene expression after FDR correction, the results were subset to only include lncRNAs. Regions were grouped if they demonstrated associations with either any of the 91 prostate, 289 TME, or the remaining 16173 lncRNAs. If multiple regions existed, the one with the lowest associated *P* value was retained.

### Differential expression analysis

The R packages EdgeR and Limma were used to perform differential expression analysis in bulk RNA-seq for the comparison between TCGA primary prostate cancer vs. WCDT mCRPC samples, as well as within mCRPC samples for *AR* region amplification vs. wild-type and *RB1* biallelic inactivation vs. monoallelic/wild-type tumor biopsies, using count data with tumor purity as a covariate^65^. For differential expression analysis in scRNA-seq data, the R function FindMarkers from the Seurat package was used with samples grouped by “liver” to assess lncRNAs associated with SCNC histology^57^. Significance thresholds for expression analysis was an absolute average log2 fold change greater than 1 and adjusted *P* value less than 10%, except for the analysis of *AR* region amplification which used an adjusted *P* value less than 25% to detect known AR-regulated lncRNAs and protein-coding genes.

### Methylation analysis

Differentially methylated regions (DMRs) for mCRPC vs. primary prostate cancer and mCRPC vs. benign prostate as well as HMRs for mCRPCs were obtained from refs. 14,66. HMRs and DMRs were annotated to lncRNAs using the nearest function^63^. HMR signatures were scored using the combined z-score approach^67^ and associated with tumor histology for 5 SCNC and 95 adenocarcinomas in ref. 14 and compared using paired *T* test.

### Single-cell RNA-seq pathway association analysis

scRNA-seq data from ref. 16 was processed as described in “Discovery and validation of cell-specific lncRNAs”^16^. Expression matrices were converted to pseudo-bulk data for each sample biopsy. The signature activity of lncRNA signatures and MSigDB Hallmark pathways for each sample was extracted using the combined z-score approach^67^ and correlated against each other using Pearson correlation coefficients (PCCs). PCCs were ranked in decreasing order to identify the top correlated pathways with lncRNAs. MSigDB Hallmark Pathways were identified in the R package msigdbr^68^.

### Bulk RNA-seq pathway association analysis

Bulk RNA-seq data for 74 mCRPC samples in the form of count data was filtered for lowly expressed genes and log CPM normalized, all using the R package edgeR^65^. Signature activity of lncRNA signatures calculated using the combined z-score approach^67^ was correlated against known pathway gene sets for genes using PCCs. MSigDB Hallmark Pathways were identified in the R package msigdbr^68^. Exhausted T-cell markers were used as described in ref. 16 and include the following: *PDCD1*,*HAVCR2*,*TOX*,*TIGIT*,*ICOS*, *FASLG*, *LAG3*, *ENTPD1*,*ITGAE*. Neuroendocrine prostate cancer gene sets were obtained from refs. 19,43. Genes upregulated in RB1 loss tumors were obtained from ref. 15.

### The area under the curve analysis

Bulk RNA-seq data for 74 mCRPC samples was processed as described in “Bulk RNA-seq pathway association analysis.” The single-cell signature of tumor histology was derived by identifying differentially expressed genes between adenocarcinomas and the small cell carcinoma sample from ref. 16 as described in “Differential expression analysis”^16^. These differentially expressed lncRNAs were used as our signature. A previously published set of 122 neuroendocrine prostate cancer lncRNAs was obtained from refs. 19,69. Signature activity of both signatures in bulk mCRPC RNA-seq data was extracted using the combined z-score approach^67^. Samples were grouped based on histology from the associated clinicopathologic metadata in ref. 13 with three mCRPC tumors labeled as “CRPC-small cell” and the remaining 71 mCRPC tumors “CRPC-adeno”. AUC analysis was performed using the roc function in the pROC R package^69^.

### Survival analysis

Bulk RNA-seq data for 74 mCRPC samples was processed as described in “Bulk RNA-seq pathway association analysis” with genomic annotations for *RB1* derived from ref. 13 Overall survival and clinical covariates were obtained from ref. 15. In total, 59 samples were matched among these datasets and used for survival analysis. For univariate analysis, overall survival from the date of diagnosis was regressed onto the signature activity calculated using the combined z-score approach^67^ on a continuous scale of *RB1* loss-associated TME (NK/T, monocyte/macrophage, neutrophil, and B lineage cells) or prostate-enriched lncRNAs using Cox-proportional hazard modeling with the survival R package^70^. Multivariate analysis was performed similarly with additional covariates for serum LDH, PSA, ALP, and hemoglobin concentrations, along with ECOG performance status and the presence of visceral metastases and enzalutamide resistance. Analysis for protein-coding gene sets associated with *RB1* deletions was done using a similar approach.

### Reporting summary

Further information on research design is available in the Nature Research Reporting Summary linked to this article.

### Supplementary information

Supplemental Figures

List of pca- and tme- lncRNAs

List of lncRNAs/protein coding genes associated with tumor progression from primary prostate cancer to mCRPC

List of lncRNAs/protein coding genes associated with AR region amplification

List of lncRNAs/protein coding genes associated with RB1 bi-allelic inactivation

List of lncRNAs/protein coding genes associated with SCNC/adenocarcinoma histology

Reporting Summary

## Data Availability

DNA methylation is available at dbGAP (phs001648) and bulk RNA-sequencing data at EGAS00001006275. All other data analyzed during this study are included in the following published articles and their supplementary information files^13–17,20,22,25,26,30,56^.

## Code availability

All analysis scripts can be found at https://github.com/ChrisMaherLab/scRNA_PCa.
