# Supplementary material for: Single cell-transcriptomic analysis informs the lncRNA landscape in metastatic castration resistant prostate cancer
Source: NPJ Genom Med. 2024 Feb 23;9:14. doi: 10.1038/s41525-024-00401-3 (PMC10891057; doi:10.1038/s41525-024-00401-3)
Supplement: Supplementary file 1 — Supplemental Figures [file 41525_2024_401_MOESM1_ESM.pdf]

Supplementary files

- Supplementary figures 1-5 (pdf):
  - Supplementary Figure 1: Expression of lncRNAs in He et al scRNA-seq dataset
  - Supplementary Figure 2: Epigenomic features are enriched and functionally important in cell-specific lncRNAs between mCRPC and primary prostate cancer
  - Supplementary Figure 3: Pca-enriched protein coding genes are associated with *AR* amplifications and treatment with enzalutamide and example of pca-lncRNA, *TP53TG1*
  - Supplementary Figure 4: TME-enriched protein coding genes are associated with *RB1* loss mCRPCs
  - Supplementary Figure 5: Example of SCNC associated lncRNA, *RMST*
- Supplementary tables 1-5 (xlsx):
  - Supplementary Table 1: List of pca- and tme- lncRNAs
  - Supplementary Table 2: List of lncRNAs/protein coding genes associated with tumor progression from primary prostate cancer to mCRPC
  - Supplementary Table 3: List of lncRNAs/protein coding genes associated with *AR* region amplification
  - Supplementary Table 4: List of lncRNAs/protein coding genes associated with *RB1* bi-allelic inactivation
  - Supplementary Table 5: List of lncRNAs/protein coding genes associated with SCNC/adenocarcinoma histology

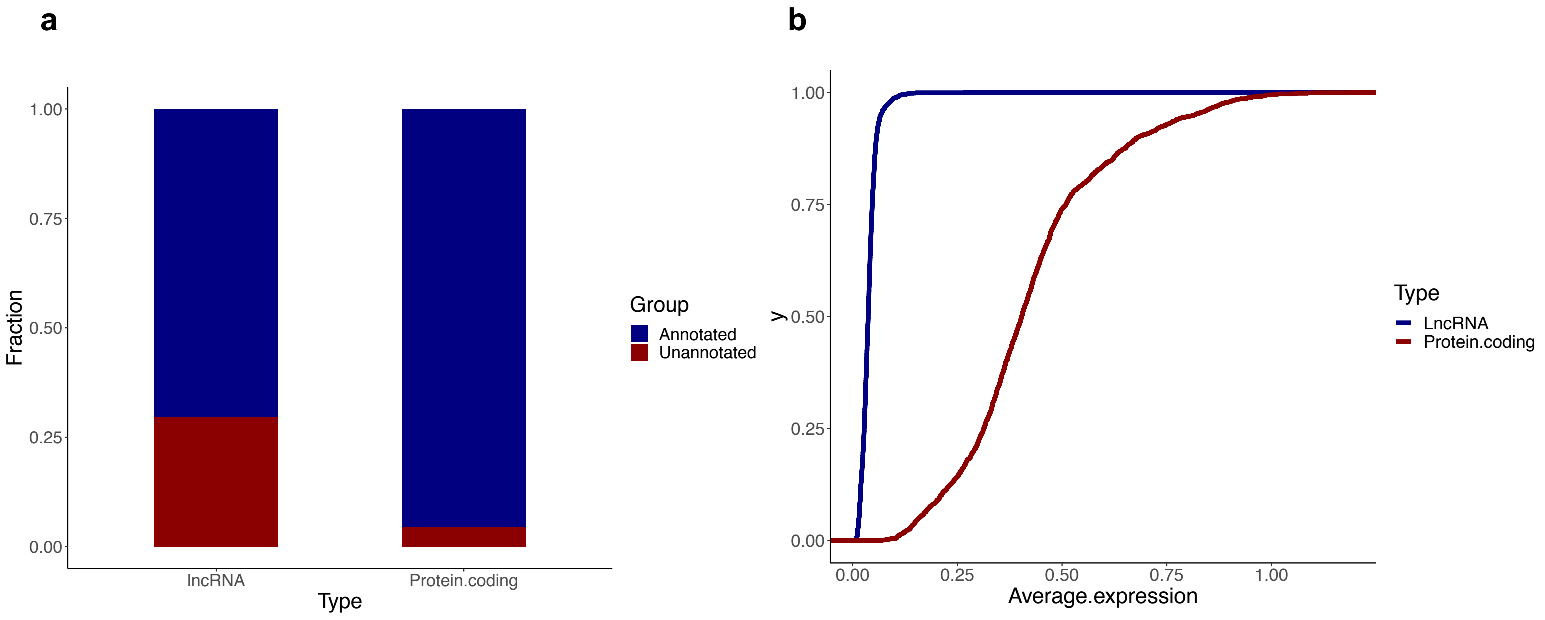

Supplementary Figure 1 Expression of lncRNAs in He et al scRNA-seq dataset: A.) Percent of annotated lncRNAs (70.4%) and protein coding genes (95.5%) in He et al. B.) Empirical CDF curves of average expression of lncRNAs and protein coding genes for the 2170 cells in the scRNA-seq dataset.

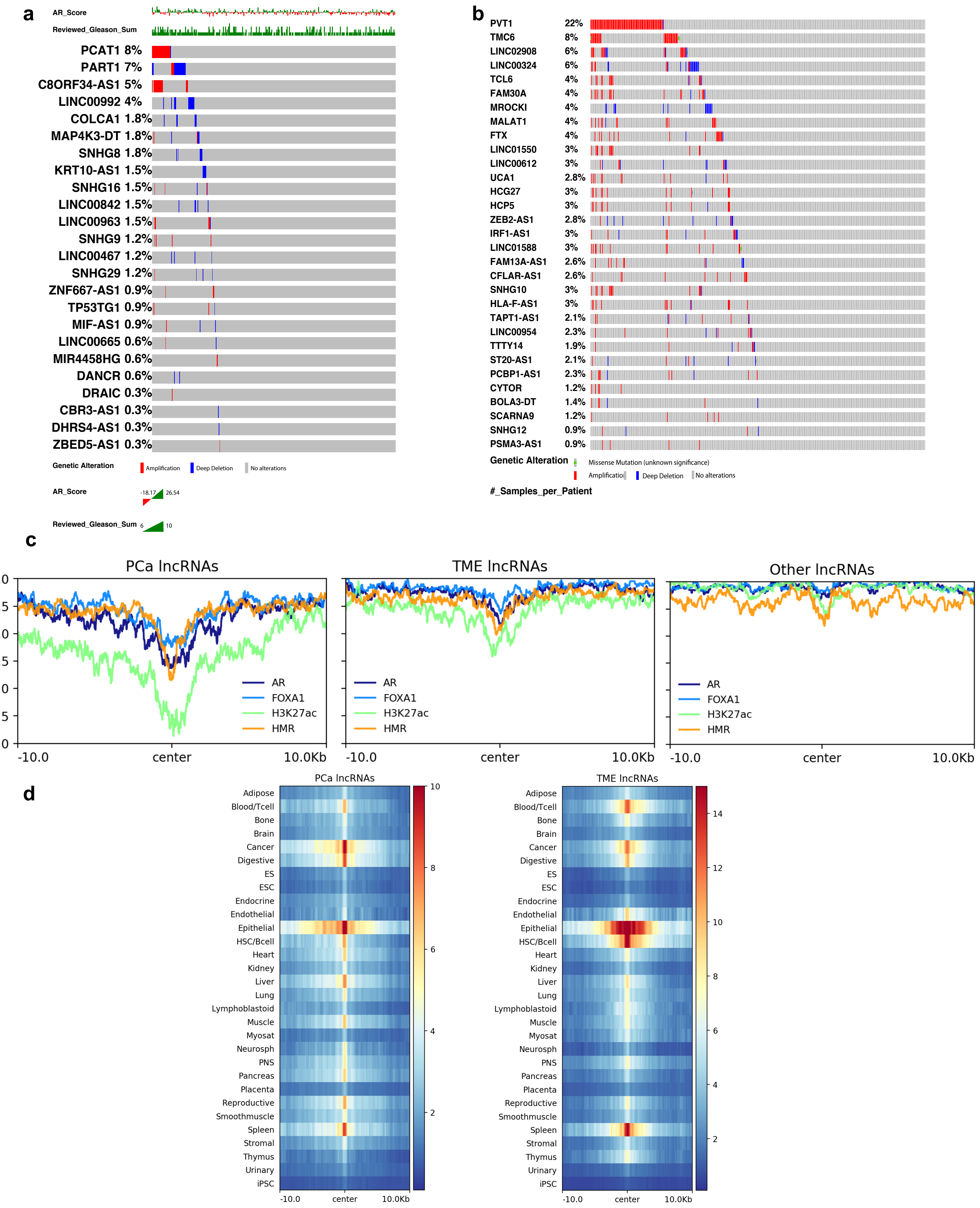

Supplementary Figure 2 Epigenomic features are enriched and functionally important in cell-specific lncRNAs between mCRPC and primary prostate cancer : A.) Mutational landscape of pca-lncRNAs with at least one somatic mutation in TCGA primary prostate cancer data B.) Mutational landscape of tme-lncRNAs in Abida et al.[22] C.) Sum of methylation differences of regulatory elements in prostate, TME, and remaining lncRNAs in hg38 downsampled to 91 genes between mCRPC and primary prostate cancer from methylation data in Zhao et al[14]. D.) Heatmaps of average logFC of EpiMap H3K27ac data in mCRPC-associated H3K27ac peaks near pca- and tme-lncRNAs.[27]

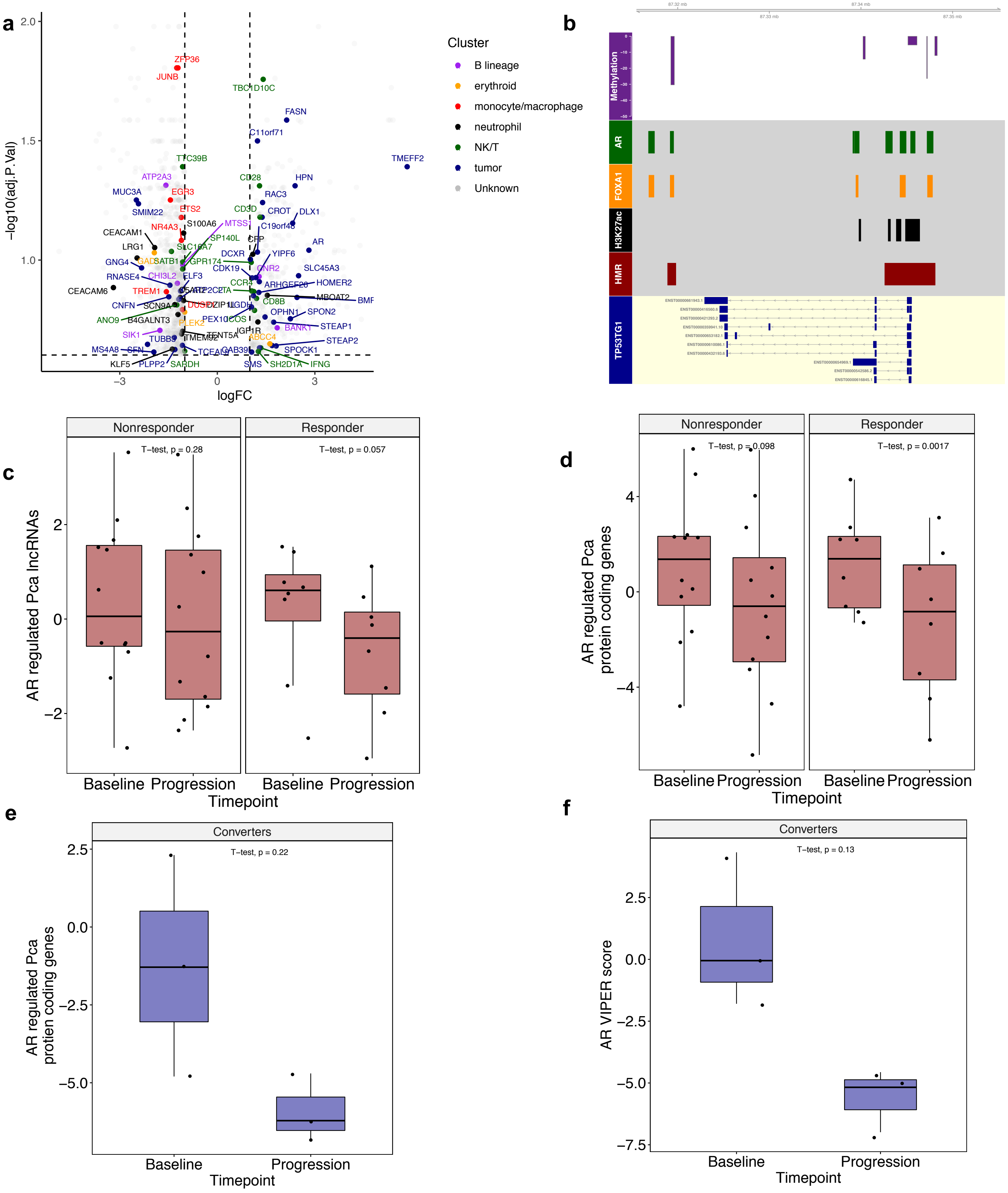

Supplementary Figure 3 Pca-enriched protein coding genes are associated with *AR* amplifications and treatment with enzalutamide: A.) Volcano plot of differentially expressed protein coding genes in tumors with *AR* region amplifications v/s wildtype in bulk RNA-seq data from n=64 mCRPCs. B.) Gene plot of TP53TG1 showing mCRPC specific HMR, H3K27ac, AR binding sites, and DMRs. C.) Expression of AR-upgregulated pca-lncRNAs in LCM RNA-seq data stratified by PSA decline (n=21) with paired T test p value above[30]. D.) Expression of AR-upgregulated pca-protein coding genes in LCM RNA-seq data stratified by PSA decline (n=21) with paired T test p value above[30]. E.) Expression of AR-upregulated pca-protein coding genes in LCM RNA-seq data for AR+/NE- to AR-/NE- converters (n=3) with paired T test p value above[30]. F.) AR VIPERs score in LCM RNA-seq data for AR+/NE- to AR-/ AR-/NE- converters (n=3) with paired

T test p value above[30].

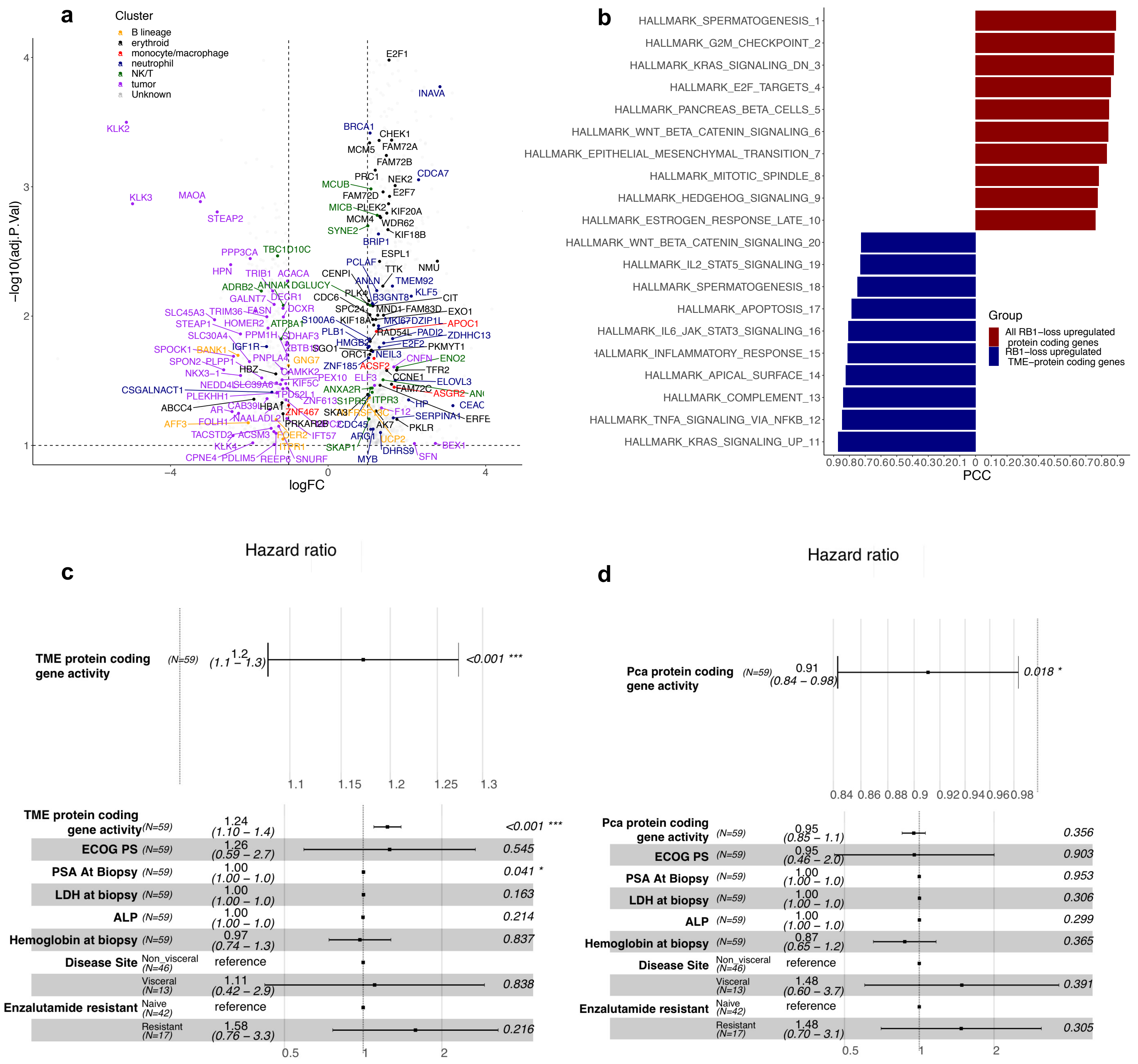

Supplementary Figure 4 TME-enriched protein coding genes are associated with *RB1* loss mCRPCs: A.) Differentially expressed protein coding genes associated with *RB1* biallelic inactivation in bulk RNA-seq data from n=64 mCRPC biopsies. Genes are clustered based on the respective enriched cell type B.) Hallmark MSigdb pathways correlated with all upregulated protein coding genes (red) and tme-protein coding genes (blue) in *RB1* deleted tumors from scRNA-seq data. C.) Forest plots of univariate (top) and multivariate (bottom) analysis of n=59 mCRPC samples with bulk RNA-seq for *RB1* loss upregulated tme-protein coding genes and overall survival. D.) Forest plots of univariate (top) and multivariate (bottom) analysis of n=59 mCRPC samples with bulk RNA-seq of *RB1* loss downregulated pca-protein coding genes and overall survival.

a

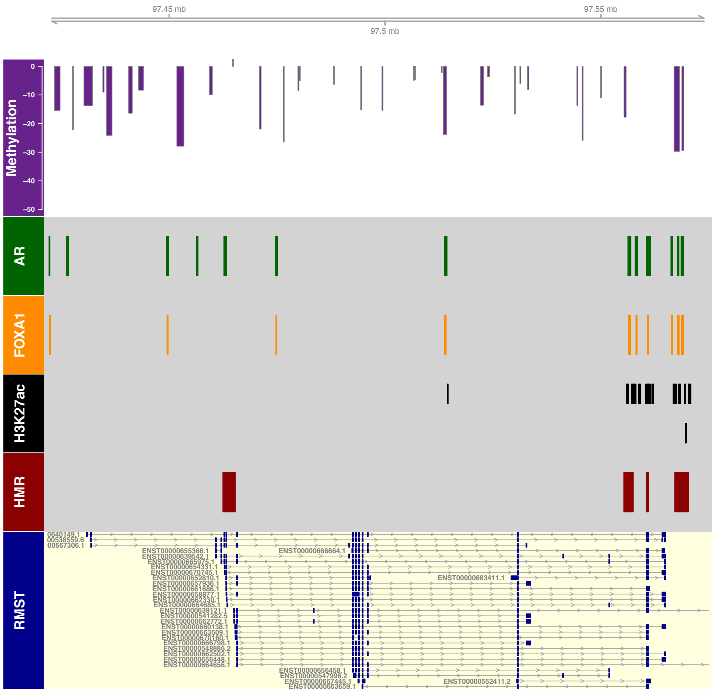

Supplementary Figure 5 Example of SCNC-associated lncRNA *RMST*: A.) Gene plot of RMST showing mCRPC specific HMR, H3K27ac, AR binding sites, and DMRs.
