# Supplementary material for: Single cell-transcriptomic analysis informs the lncRNA landscape in metastatic castration resistant prostate cancer
Source: NPJ Genom Med. 2024 Feb 23;9:14. doi: 10.1038/s41525-024-00401-3 (PMC10891057; doi:10.1038/s41525-024-00401-3)
Supplement: Supplementary file 7 — Reporting Summary [file 41525_2024_401_MOESM7_ESM.pdf]

nature portfolio

Corresponding author(s): Christopher Maher, Debanjan Saha
Last updated by author(s): Sep 27, 2023

Reporting Summary

Nature Portfolio wishes to improve the reproducibility of the work that we publish. This form provides structure for consistency and transparency in reporting. For further information on Nature Portfolio policies, see our Editorial Policies and the Editorial Policy Checklist.

Statistics

For all statistical analyses, confirm that the following items are present in the figure legend, table legend, main text, or Methods section.

n/a	Confirmed	
☐	☒	The exact sample size (*n*) for each experimental group/condition, given as a discrete number and unit of measurement
☐	☒	A statement on whether measurements were taken from distinct samples or whether the same sample was measured repeatedly
☐	☒	The statistical test(s) used AND whether they are one- or two-sided<br>*Only common tests should be described solely by name; describe more complex techniques in the Methods section.*
☐	☒	A description of all covariates tested
☐	☒	A description of any assumptions or corrections, such as tests of normality and adjustment for multiple comparisons
☐	☒	A full description of the statistical parameters including central tendency (e.g. means) or other basic estimates (e.g. regression coefficient) AND variation (e.g. standard deviation) or associated estimates of uncertainty (e.g. confidence intervals)
☐	☒	For null hypothesis testing, the test statistic (e.g. *F*, *t*, *r*) with confidence intervals, effect sizes, degrees of freedom and *P* value noted<br>*Give P values as exact values whenever suitable.*
☒	☐	For Bayesian analysis, information on the choice of priors and Markov chain Monte Carlo settings
☒	☐	For hierarchical and complex designs, identification of the appropriate level for tests and full reporting of outcomes
☐	☒	Estimates of effect sizes (e.g. Cohen's *d*, Pearson's *r*), indicating how they were calculated

Our web collection on statistics for biologists contains articles on many of the points above.

Software and code

Policy information about availability of computer code

Data collection	DNA methylation are available at dbGAP (phs001648) and bulk RNA-sequencing data at EGAS00001006275. All other data analyzed during this study are included in the published articles and their supplementary information files as cited in the manuscript. These included single cell sequencing data from He et al. (lymph node, bone and liver metastases, n=14), Kfoury et al. dataset (bone metastases, n=9), Chen et al dataset (lymph node metastases, n=2) , and Song et al (primary tumors, n=11). Whole genome sequencing data was analyzed from Abida et al. (n=444) and Quigley et al (n=101). DNA methylation data (n=100) was obtained from Zhao et al. at dbGAP (phs001648) and bulk RNA-sequencing data (n=74) at EGAS00001006275. Overall survival data with clinical covariates for these mCRPC samples was downloaded from Chen et al. (n=100). Laser-capture microdissection RNA-sequencing data with associated metadata was obtained from Westbrook et al. (n=22). Chromatin Immunoprecipitation (ChIP)-sequencing datasets for AR, FOXA1, and H3K27ac were obtained from Severson et al. (n=4). RNA sequencing count data from TCGA primary prostate cancer samples (n=333) and normal adjacent tissue (n=50) and the associated metadata were downloaded from Abeshouse et al.
Data analysis	All analysis scripts can be found on https://github.com/ChrisMaherLab/scRNA_PCa .

For manuscripts utilizing custom algorithms or software that are central to the research but not yet described in published literature, software must be made available to editors and reviewers. We strongly encourage code deposition in a community repository (e.g. GitHub). See the Nature Portfolio guidelines for submitting code & software for further information.

Data

Policy information about availability of data

All manuscripts must include a data availability statement. This statement should provide the following information, where applicable:

- Accession codes, unique identifiers, or web links for publicly available datasets
- A description of any restrictions on data availability
- For clinical datasets or third party data, please ensure that the statement adheres to our policy

DNA methylation are available at dbGAP (phs001648) and bulk RNA-sequencing data at EGAS00001006275. All other data analyzed during this study are included in the published articles and their supplementary information files as cited in the manuscript.

Research involving human participants, their data, or biological material

Policy information about studies with human participants or human data. See also policy information about sex, gender (identity/presentation), and sexual orientation and race, ethnicity and racism.

Reporting on sex and gender	Reporting on sex and gender is described in the original publications referenced in this manuscript.
Reporting on race, ethnicity, or other socially relevant groupings	Reporting on race and ethnicity is described in the original publications referenced in this manuscript.
Population characteristics	Reporting on population characteristics is described in the original publications referenced in this manuscript.
Recruitment	Participant recruitment is described in the original publications referenced in this manuscript.
Ethics oversight	Reporting on ethics oversight is described in the original publications referenced in this manuscript.

Note that full information on the approval of the study protocol must also be provided in the manuscript.

Field-specific reporting

Please select the one below that is the best fit for your research. If you are not sure, read the appropriate sections before making your selection.

☒ Life sciences ☐ Behavioural & social sciences ☐ Ecological, evolutionary & environmental sciences

For a reference copy of the document with all sections, see nature.com/documents/nr-reporting-summary-flat.pdf

Life sciences study design

All studies must disclose on these points even when the disclosure is negative.

Sample size	Sample size determinations are described in the original publications referenced in this manuscript.
Data exclusions	Data exclusion determinations are described in the original publications referenced in this manuscript.
Replication	Multiple datasets were used for validation in this study. Cell specific lncRNAs were derived from He et al. (lymph node, bone and liver metastases, n=14) and validated in the Kfoury et al. dataset (bone metastases, n=9) , Chen et al dataset (lymph node metastases, n=2) , and Song et al (primary tumors, n=11).
Randomization	In the analysis of lncRNAs associated with prostate cancer progression and genomic status, tumor purity was controlled for between datasets to account for batch effects. In the analysis of lncRNAs associated with overall survival,multivariate analysis was performed similarly with additional covariates for serum LDH, PSA, ALP, and hemoglobin concentrations, along with ECOG performance status and the presence of visceral metastases and enzalutamide resistance.
Blinding	Group allocation was performed in the original publications referenced in this manuscript and so investigators in this study were blinded.

Reporting for specific materials, systems and methods

We require information from authors about some types of materials, experimental systems and methods used in many studies. Here, indicate whether each material, system or method listed is relevant to your study. If you are not sure if a list item applies to your research, read the appropriate section before selecting a response.

Materials & experimental systems

n/a	Involved in the study	
☒	☐	Antibodies
☒	☐	Eukaryotic cell lines
☒	☐	Palaeontology and archaeology
☒	☐	Animals and other organisms
☒	☐	Clinical data
☒	☐	Dual use research of concern
☒	☐	Plants

Methods

n/a	Involved in the study	
☒	☐	ChIP-seq
☒	☐	Flow cytometry
☒	☐	MRI-based neuroimaging
